# Multimetal bioremediation and biomining by a combination of new aquatic strains of *Mucor hiemalis*

**DOI:** 10.1038/s41598-019-46560-7

**Published:** 2019-07-16

**Authors:** Enamul Hoque, Johannes Fritscher

**Affiliations:** Helmholtz Zentrum München - German Research Center for Environmental Health (GmbH), Institute of Groundwater Ecology, 85764 Neuherberg, Germany

**Keywords:** Environmental biotechnology, Environmental chemistry

## Abstract

Here we describe a unique microbial biotechnology for simultaneous bioremediation and biomining of twelve ionic metals overcoming the obstacles of multimetal toxicity to microbes. After a thorough search of key microorganisms in microbiomes of many sulfidic springs in Bavaria (Germany) over an area of 200 km^2^, we found three new strains EH8, EH10 and EH11 of *Mucor hiemalis* physiologically compatible and capable of multimetal-remediation and enrichment. We combined the multimetal-resistance, hyper-accumulation and elicitation power of EH8, EH10 and EH11 to develop a novel biotechnology for simultaneous removal, fractionation and enrichment of metal ions. As a first step we showed the intracellular fixing and deposition of mercury as nanospheres in EH8’s sporangiospores. Scanning Electron Microscopy-Energy-Dispersive X-Ray analysis revealed binding and precipitation of other applied metal ions as spherical nano-particles (~50–100 nm) at the outer electro-negative cellwall-surface of EH8, EH10 and EH11 sporangiospores. Microbiomes, germinated spores and dead insoluble cellwalls of these strains removed >81–99% of applied Al, Cd, Co, Cr, Cu, Hg, Ni, Pb, U, and Zn simultaneously and furthermore enriched precious Ag, Au and Ti from water all within 48 h, demonstrating the potential of new biotechnologies for safe-guarding our environment from metal pollution and concentrating precious diluted, ionic metals.

## Introduction

Increasing global demand and use of various metals/metalloids leads to their accumulation in the environment and thereby simultaneously increases the risk of multimetal-toxicity to various organisms. In higher organisms accumulated heavy metals can damage nerves, liver, bones, etc., as well as block functional groups of enzymes and cause cancer^1^. This metal accumulation is mainly due to various industrial processes such as; metal production, mining, electroplating, nuclear power generation, municipality waste incineration and by manufacture of a myriad of metal-containing electronic components^2^. Toxic metals/metalloids may even be mixed with recalcitrant and/or radioactive organic pollutants, e.g. Cu and biphenyl^3^, which can increase the severity of metal toxicity and problems of remediation. Controlling this pollution now represents a large challenge for our society. The removal and recycling of toxic metals or metalloids, particularly from water phase, deserves our special attention because metal-contaminated materials like soil, sediment and gaseous emissions can be washed-off with water, so that ultimately metal contaminants are highly concentrated in, e.g., waste water (nuclear, metallurgical, mining) and landfill leachates. Several technologies have been described previously for the removal of toxic metals or metalloids from water, e.g. flocculation^4^, adsorption^4^, bioaccumulation by live microbial cells^5^, immobilization^6,7^ and reverse osmosis^4^. Existing technologies for metal removal from aqueous phase are either too costly, e.g. by reverse osmosis^4^, or they create excessive additional waste.

In contrast to *ex-situ* physical-chemical methods, *in-situ* natural enrichments of metal ions may take place due to physical-chemical and/or biological processes. Natural hydrothermal conditions, e.g. at temperatures above 60 °C, can enhance oxidative-reductive complexation and precipitation processes of metal ions in water, but have high energy requirements. Therefore, there is an increasing interest in biotechnology for the development of low-cost, low energy-consumption and effective bioremediation^8^ and biomining^9^. *Ex-situ* or *in-situ* metal/metalloid removal by microbial biosorption^2^ and enzyme-mediated metal precipitation at cell surfaces^10^ could be cheap alternatives for treating metal-contaminated wastewater and/or water purification. So far, only a limited number of natural microorganisms have been found to show biosorption properties for a few toxic metals/metalloids, whereby they required very stringent culture conditions, e.g. concerning C- and N-source^11^ and an optimum pH^12^. Therefore, genetically modified microorganisms (GMO), e.g. *Ralstonia eutropha*, *Escherichia coli* and *Saccharomyces cerevisiae*, were developed to improve microbial hyper metal-binding capacity/accumulation by proteins^13^, however their usage faces serious legal restrictions in field applications due to the risk of uncontrolled GMO release and horizontal gene transfer. Microbial volatilization of metals/metalloids from such metal wastes creates additional safety problems during handling. The key obstacle in the development of suitable microbial biotechnology for multimetal bioremediation and biomining is the lack of knowledge about avoiding extreme multimetal toxicity to microorganisms. In this context it is worth mentioning that microbial removal of toxic metal mixes of nickel and cadmium from water phase was very difficult because of high multimetal toxicity to *Clostridium thermoaceticum* even at a concentration of only 1 mM^14^. In addition, microbial tolerance to toxic metals differs, e.g. the following order of decreasing tolerance of microbes to cadmium, whereby the Cd toxicity increased multi-fold at pH 8−9, was found: actinomycetes > eubacteria > gram-negative bacteria > gram-positive bacteria^15^. As such large differences in toxic metal removal exist, for example 90% of applied cadmium was shown to be removed by a new strain of *Pseudomonas aeruginosa* in a defined aerobic culture^16^, whereas only 12% of applied cadmium was removed by another *Pseudomonas aeruginosa* strain ATCC 14886^17^. Even genetically modified *E. coli* with a surface display of metal trapping peptides was shown to mainly bind Cd^2+^, and to lesser extents Cu^2+^ and Zn^2+^ ^18^, highlighting the contemporary limitations of bacterial toxic multimetal removal.

An alternative biotechnology for the removal of toxic metals could be the use of fungi^19^ and fungal microbiomes (biofilms)^5^. The terrestrial fungus *Penicillium ochrochloron* has been used as a biotrap for the removal of Cu^2+^ and other toxic metal ions from aqueous solutions and surrogate waste waters^2^. Some terrestrial fungal biomasses have been chemically pre-treated to improve their metal remediation function^20,21^. Several strains of the yeast *Rhodotorula* showed more resistance to Cd than to the heavy metals Ag, Co, Hg and Ni. However, there was no correlation between class of soil fungus and tolerance to cadmium^15^. Due to the high severity of multimetal toxicity in fungi, until now, as far as known, a maximum of 2–5 toxic metals like Pb, Cd, Cr, Cu, Mn, Ni, Zn were effectively removed at a time by a single terrestrial fungus, e.g. *Aspergillus fumigatus*, *Aspergillus niger*, *Aspergillus terreus*, *Macrophomina phaseolinia*, *Penicillium* sp., *Rhizupus stolonifera*, *Trichoderma viridae*, *Trichoderma longibrachiatum*^22–25^. Although the terrestrial fungi *Rhizopus* and *Trichoderma* showed high resistance to a range of heavy metals, such as Cd, Cu, Pb and As, they were not assayed in the same experiment^26^. Viable cells of the yeast *Saccharomyces cerevisiae* removed five metals Cu, Cr, Cd, Ni and Zn from electroplating effluents, but only after glucose-pretreatment^27^.

In contrast to terrestrial fungi, aquatic fungi and their natural microbiomes at hydro-terrestrial interfaces have exhibited high metal enrichments and resistance^5,﻿28﻿,29^ and protected the unique natural consortia by enriching toxic multimetals from competitive successions of other organisms, e.g. by, micro- and macro algae. In co-presence of harmful multimetals and organic toxins, some fungi may be especially useful because they possess an additional repertoire of enzymes for the detoxification of organic toxins^2^. In particular the aquatic filamentous fungus *Mucor hiemalis* EH5 was shown to remove various organic pollutants, especially in fungal dual cultures^30^, as well as cyanobacterial toxins^31,32^, furthermore its natural microbiome enriched some metal ions^28^. The strains of aquatic *M. hiemalis* could be useful for outdoor metal bioremediation under extreme natural conditions, as EH5 was found to thrive *in situ* in hostile conditions, i.e. cold sulfidic, reducing and/or nearly anoxic aquatic milieu in presence of diverse metals in the natural microbiomes of sulfidic springs^28^. Alternatively to using live fungal mycelium biomass^11^ and germinated spores^5^ for intracellular accumulation and/or biosorption and precipitation, fungal hyphal cell walls can be used, e.g. for chromium removal by terrestrial *M. hiemalis* cell walls^11^ or for lead removal by *Penicillium chrysogenum* cell walls^33^. So far, few individual live fungi or dead fungal cell walls have been used for the removal of only a few toxic metals from water^11,33^, as the live fungal mix (species/strain) may be mutually antagonistic during growth. This could be the reason why the successful use of fungal species/strain combinations for biotechnological applications was, until now, lacking.

Through our continued search for biotechnologically relevant fungi, we recently succeeded in isolating the novel aquatic strains of *Mucor hiemalis* EH8 and EH11 from the microbiomes of cold sulfidic spring waters with extraordinary metal/metalloid bioaccumulation capacity^5,29^. They were found to be crucial in the fungal-bacterial microbiomes of sulfidic-sulfurous springs for the hyper-accumulation of metals. The purpose of our study was to show how to develop suitable biotechnology for multimetal remediation, fractionation and enrichment based on; 1. Natural observations, ecological considerations, microbial biodiversity and successions; 2. Identification of key aquatic fungal strains (*Mucor hiemalis* EH8, EH 10 and EH11) in microbiomes of sulfidic spring water; and 3. Inhibition/toxicity assays and use of compatible strains (EH8, EH10 and EH11), e.g. as (a) Activated live sporangiospores, (b) live microbiomes and (c) Purified cell walls from solvent-killed single, and mixed-grown fungal strains^5,29^ for the simultaneous removal of diverse toxic metals and enrichment of precious metals. Here we describe in detail the testing of physiological tolerances of EH8, EH10 and EH11 in co-cultures, the isolation and preparation of mixed fungal materials (microbiomes, germinated spores and dead insoluble cell walls), the quantitative kinetics of simultaneous metal removal, the localization of metal precipitation by scanning electron microscopy (SEM) and the analysis of composition of metal precipitates by energy-dispersive x-ray (EDX) microanalysis. This is the first report about the application of triple fungal co-cultures using EH8, EH10 and EH11 for simultaneous multimetal remediation, fractionation and hyper-enrichment (e.g. for biomining), with up to 99% efficiency for some precious metals, from water phase.

## Results

### Hydro-geochemistry of sulfidic springs and microbial succession

Table 1 shows the chemical and physical parameters of the sulfidic springs Marching, Quarzitwerk and Künzing, from which the *M. hiemalis* strains EH8, EH10 and EH11 were isolated, respectively. In an area of about 200 km^2^ we investigated the multimetal enrichment, microbial diversity and succession of microorganisms in microbiomes (biofilms) of 18 different sulfidic springs by metal screening assays, phase contrast and electron microscopy, molecular biological techniques and morphological comparison. Three strains of *Mucor hiemalis* (EH8, EH10 and EH11) were found to be crucial in their respective natural microbiomes for accumulation of diverse toxic metals, as the metal removal or enrichments by pure cultures matched the values for their respective natural microbiomes (Table S1). Bryophyta and macrozoobenthos typical of sulfidic springs were absent in the microbiomes of these springs, but *Archaea*, *Bacteria*, ciliates and diatoms were detected^5,29^.

**Table 1:** Chemical and physical features of Marching, Quarzitwerk (Murnauer Moos) and Künzing spring waters as compared to control spring Teugn (September 2003).

Component Parameters/Springs	Unit	Marching	Quarzitwerk^a^	Künzing	Teugn
Spring discharge	L min^−1^	120	180	210	150–200
**Temperature**	°C	10.2–10.6	10.1–11.4	**18.9–19.0**	12.4–12.7
**Electrical Conductivity**	μS cm^−1^	623–672	867–966	**1310–1320**	665–697
pH		5.9–6.5	5.1–6.3	7.3–7.5	4.7–6.4
**Redox Potential (E**_**h**_ **)**	mV	−185 to –173	−106 to −97	−**253 to** −**241**	−215 to −192
**Oxygen**	mg l^−1^	1.3–1.8	0.4–1.1	**≤0.1**	0.7–1.2
H_2_S	mg l^−1^	<1	n. m.	≤1	n. m.
**Cations and total metal ions:**					
**Na** ^**+**^	mg l^−1^	6.0–8.2	5.5–5.7	**164.4–398.5**	50.7–54.7
K^+^	mg l^−1^	0.7–1.1	0.70–1.1	10.1–10.5	6.5–6.9
Mg^2+^	mg l^−1^	31.3–31.5	17.7–17.9	8.3–9.4	22.7–22.9
Ca^2+^	mg l^−1^	81.2–85.9	109.4–120.5	12.6–47.3	72.7–74.2
**Mn** ^**2+**^	mg l^−1^	4.5–4.9	**44.8–270.2**	2.5–2.7	7.6–9.5
Ba^2+^	μg l^−1^	15.5–20.4	16.5–32.4	25.8–45.5	47.0–58.2
Total Co	μg l^−1^	<1	<1	<1	0–16.0
**Total Cu** ^**2+**^	μg l^−1^	**3.2–6.0**	0–2.7	0–14.4	1.1–7.6
Total Fe	μg l^−1^	1.9–7.8	8–16.7	15.6–20.2	9.8–16.7
Li^+^	μg l^−1^	6.2–7.9	2.3–3.0	202.0–279.4	41.8–49.5
**Sr** ^**2+**^	μg l^−1^	78.9–117.2	213.1–333.8	**286.1–1005.4**	78.9–117.2
**Zn** ^**2+**^	μg l^−1^	**367.1–437.6**	163.2–237.2	n. d.	265.2–288.4
**Anions:**					
SH^−^	mg l^−1^	0.4–0.6	1.1–1.3	0.7–0.9	1.1–1.3
**Cl** ^**−**^	mg l^−1^	11.5–12.1	0.6–2.5	**320.1–354.5**	25.5–26.3
Br^−^	mg l^−1^	n. d.	n. d.	0.2	n. d.
J^−^	mg l^−1^	n. d.	n. d.	0.2	n. d.
**NO**_**3**_^**−**^	mg l^−1^	**0.1–1.1**	**0.1–1.1**	n. d.	0.2
SO_4_^2−^	mg l^−1^	36.6–37.5	6.2–8.7	n. d.	16.1–26.2
HCO_3_^−^	mg l^−1^	360.0–360.5	434.7–434.9	572.00	420.6–420.9
DOC*	mg l^−1^	1.1–1.5	6.4–8.9	**n. d**.	0.4–1.1
**Gas Bubbles:**					
**CH**_**4**_ **(emission)** ^**b**^	m^3^ d^−1^	n. d.	—	**7**	—

The contrasting parameters of these three springs as compared to the control spring Teugn are highlighted in bold font. Abbreviations are: ^a^disappeared in the year 2004, *DOC: dissolved organic carbon, ^b^Carle^58^.

### Adaptation and evolution of fungal strains that hyper-accumulate toxic metals

**Figure 1:**
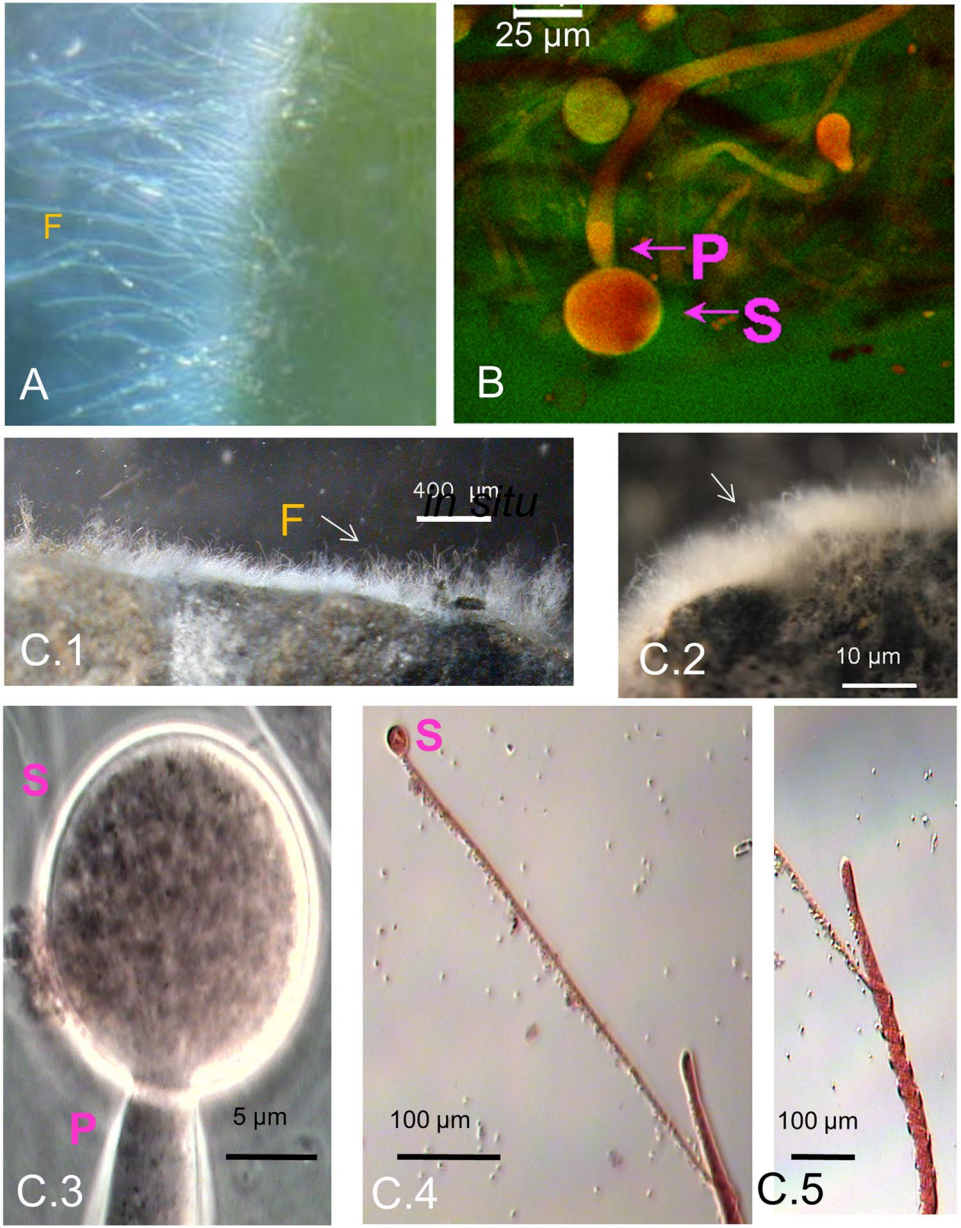
Morphology of selected *Mucor hiemalis* strains from sulfidic spring water microbiomes. (**A**) Adaptation and *in situ* morphology of mercury-accumulating *M. hiemalis* strain EH8 (F) attached to a moss leaf. (**B**) Laser scanning and stereo microscopy revealed similar morphology (S: sporangium, P: sporangiophore) of *M. hiemalis* EH5, EH8 and EH10 strains. (**C**) Detailed morphology and adaptation of aquatic *M. hiemalis* strains. The brush-like morphology of EH11’s *in situ* microbiome fixed on rock (F, C.1) from the methane–emitting salty sulfidic environment of Künzing spring and of EH8’s *ex-situ* grown microbiome fixed on expanded clay spheres (C.2) is visible. EH11 (C.3, sporangium S with sporangiophore P) showed spring-like hyphal morphology (C.4 and C.5) due to adaptation to bubbling methane. However, the spring-like hyphal morphology of EH11 disappeared after further cultivations on solid malt extract-agar medium.

Several strains of *M. hiemalis* were found in the special habitats of sulfidic springs and showed unusual hyper-accumulation capacity for different metals, similar to that of their respective microbiomes (Table S1). Their hyper-metal accumulation properties could be due to adaptive pressure and acceleration of evolution triggered by extreme sulfidic spring water environments (see supplement). We have already reported the adaptation of EH8 to mercury stress and its ecological functions^5^. Several strains of *Mucor hiemalis* from cold sulfidic spring water microbiomes were isolated and assayed for their physiological compatibility as well as for their metal removal functions (see below). EH8’s filamentous microbiome fixed to a moss leaf is shown in Fig. 1A^5^. EH10 from Quarzitwerk spring microbiome was morphologically similar to EH5 and EH8 (Fig. 1B).

EH11 was isolated from the sessile microbiome exhibiting a brush-like morphology fixed on rock in the anaerobic methane-emitting sulfidic Künzing spring (Fig. 1C.1). Similar to EH11, EH8 also had a brush-like appearance and grew on expanded clay (Fig. 1C.2). The strain EH11 showed spiral hyphae in response to bubbling methane and unusual anaerobic metabolism and asexual reproduction^29^.

### Fungal inhibition/toxicity assay

**Figure 2:**
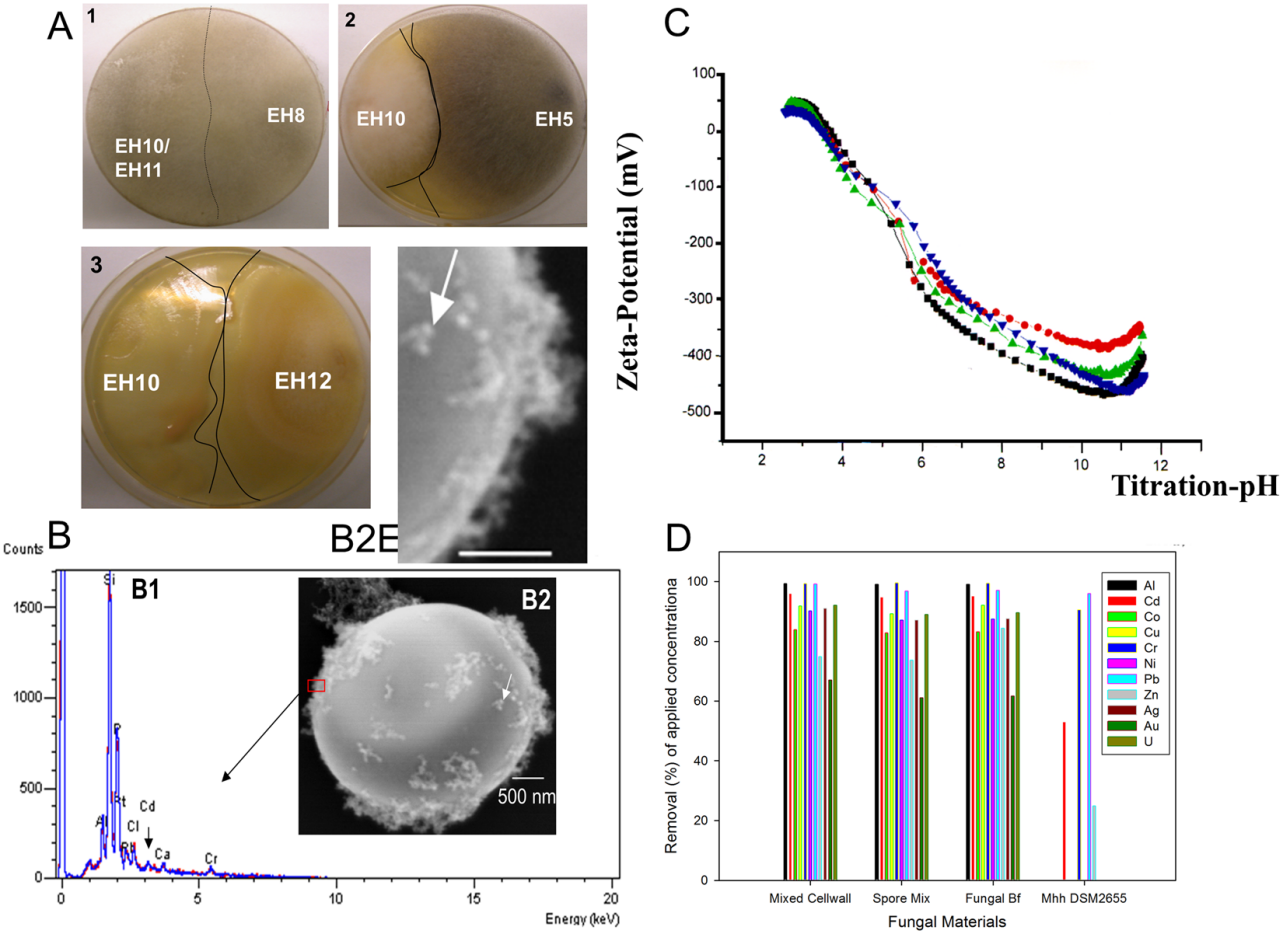
Inhibition/toxicity tests of *M. hiemalis* strains, detection of chitin, metal removal capacity and surface potential. (**A1-3)** Inhibition/toxicity tests of aquatic fungal strains, A1. Non-existence of demarcation lines between mycelial fronts showing absence of antagonistic inhibitory reactions among EH8, EH10 and EH11 when they were challenged against each other or grown together in the same plate, A2. Demarcation lines and discolouration indicating antagonistic reactions between EH5 and EH10, and A3. Demarcation lines and oily droplet formations illustrating antagonistic reactions between EH10 and EH12. (**B-D)** Relationship between metal binding and zeta-potential of the sporangiospore’s cell surface. (**B)** EDX detection of metals bound to the surfaces of sporangiospores, B1. EDX-detection of Al, Pb, Cd, Cr and P at a spot (red rectangle) on the outer surface of the sporangiospores (B1), B2 and B2E (enlarged). Formation of ca. 50–100 nanometer-sized particles (nanospheres; see white arrows) at the outer cell surfaces of sporangiospores following 48 h incubation in metal salt solutions (pH ∼7). (**C**) Zeta-potential of aquatic *M. hiemalis* sporangiospores after germination (1–3 cell stages) depending on nutrient conditions of incubation medium (red circle, C-limited medium; green triangle, C- and N-enriched medium; downward blue triangle, N-limited medium; black square, groundwater control) after 48 h incubation at approx. 30 °C. (**D**) Removal of metals by dead insoluble cell walls, live spore mix and live microbiomes (Fungal Bf) of strains EH8, EH10 and EH11 in comparison to the control terrestrial fungus DSM 2655. Horizontal bar in B2 and 2B2E indicates scale of 500 nm.

In fungal inhibition/toxicity assays^34^ the strains EH8, EH10 and EH11 were not mutually antagonistic (Fig. 2). No visible demarcation line between the dual culture mycelium fronts of EH8 and EH10/EH11 was found, suggesting they are physiologically tolerant to each other (Fig. 2A.1). Other dual cultures of aquatic *Mucor hiemalis*, e.g. EH5^28^ and EH10, and EH10 and EH12, reacted antagonistically to one another, as shown by e.g. formation of demarcation lines between mycelium fronts, release of oily drops at the hyphal tips and color changes in response to the presence of antagonistic strains (Fig. 2A.2–A.3). Purified viable germinating sporangiospores of aquatic *M. hiemalis* strains showed, as expected, strong presence of chitin that could be recognized and visualized as tiny anti-chitin stain spheres on the cell walls, as detected by fluorescence microscopy of anti-chitin fluorescein-labeled antibody (Fig. S2).

### Zeta-potential of cell surface, hyper-metal accumulation and precipitation

Acid-base titration of the aquatic *M. hiemalis* sporangiospores did not show any distinct pK-values in the investigated pH range suggesting no domination of weak acids or bases (Fig. 2A). The zeta-potential of spore cell surfaces varied depending on nutrient/mineral contents of the incubation medium. Surprisingly, a groundwater-like environment with low mineral and nutrient contents was shown to increase the electro-negativity of the sporangiospores in the

pH range 6–10, if they were pre-germinated in groundwater (Fig. 2A). Contrastingly, under a mineral-rich medium with high C- or N-contents^35^ the zeta potential of sporangiospores’ surfaces lost ca. 10% of the electro-negativity at pH 7, mainly due to the favored pre-occupation of some electronegative sites on the spore surfaces by the metal cations supplemented to the fungal growth medium. The surface potentials of the spores showed rapidly increasing electro-negativity (<−100 mV down to −480 mV) with increasing pH, indicating strong electrical attraction of aquatic *M. hiemalis* spore cell walls towards the positively charged metal cations.

### Production of spherical metal nano- and micro-particles by activated and elicited sporangiospores

When a mixture of metal ions came into contact with cell surfaces of the activated sporangiospores of EH8, EH10 and EH11, metal spheres were readily produced. SEM showed the adsorption and precipitation of approx. 50–100 nm-sized spherical metal nanoparticles on the cell walls following 48 h incubation of mixed EH8, EH10 and EH11 activated sporangiospores in a metal mix solution (Fig. 2B1,B2,B2E). This was in accordance with the metal cation-attractive forces of the strongly electro-negative zeta-potential of spores’ outer surfaces (Fig. 2C). EDX microanalysis localized the outer cellular surface of sporangiospores as the site of biosorption and precipitation of diverse metal nano-particles, e.g. Al, Pb, Cd and Cr, and showed the presence of P at the same spot, indicating selective spherical metal precipitations at the electro-negative reducing chitinised centers (Fig. S2) and phosphate-ligand sites of the cell surfaces (Fig. 2B2). This result is similar to the phenomenon of copper phosphate precipitation in *Penicillium ochrochloron* ATCC 36741^2^. The metal elimination power of the dead mixed insoluble cell walls, the spore mix and the mixed microbiomes of EH8, EH10 and EH11 is shown in Fig. 2D. Results showed a similar metal removal capacity of the microbiomes, the spore mix and the mixed cell walls.

### Removal of metal ions by mixed grown EH8, EH10 and EH11

The first use of activated EH8 sporangiospores eliminated Hg(II), as well as some other metal ions, from water by intracellular accumulation (Table S1)^5^. Further results of metal removal by the microbiomes and corresponding fungi are summarized in Table S1. Metal resistance and accumulation assays showed that EH8 of the Marching spring microbiome interfacing moss leaves (Fig. 1A) hyper-accumulated Al (90%), Cr(III) (99%), Ni (86%) and U (89%) at the cell surfaces as well as Hg (99%)^5^ intracellularly. EH10 (Fig. 1B) from the Quarzitwerk spring microbiome additionally hyper accumulated Cd (91%), Cu (85%), Pb (93%), and Zn (71%) (Table S1). In contrast, anaerobic EH11 with its unusual spring-like hyphal adaptation (Fig. 1C.3,C.5) from the microbiome of methane-emitting salty sulfidic Künzing spring hyper-accumulated Al (98%), Cr(III) (89%), Cu (87%), Pb (97%), Ni (82%) and Zn (83%)^29^. If EH8, EH10 and EH11 were grown together, they became elicited and additionally enriched precious metal ions, e.g. Ag, Au and Ti. Therefore, the great potential of these mutually tolerant strains, EH8, EH10 and EH11, was demonstrated for the development of low-cost biotechnology for multimetal bioremediation and/or biomining.

### *In situ* generation of diverse element particles by microbiomes

**Figure 3:**
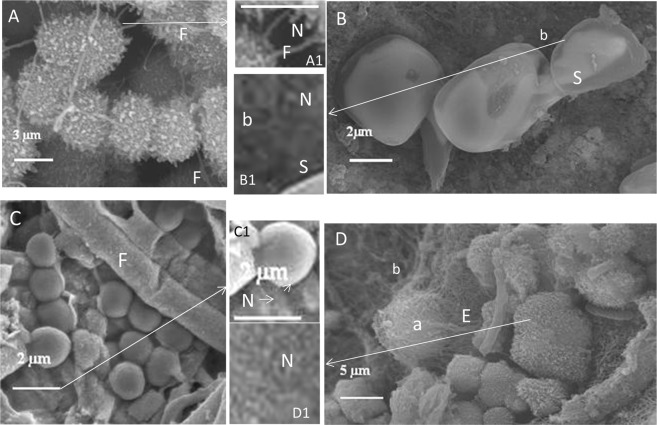
Precipitation of nanometer- to micrometer-sized mineral particles in sulfidic spring microbiomes. (**A**) Aggregates of micrometer-sized spherical iron sulfide particles with fungal hyphae (F), A1 (zoomed view) Fungal hyphae (F) with nanospheres (N). (**B**) Sulfur crystals (S) with bacteria (b), B1 (zoomed view) Sulfur crystal (S) with Bacteria (b) and nanospheres (N). (**C**) Spherical iron aggregate colloids with fungal hyphae (F), C1 (zoomed view) Iron colloid with nanospheres (N). (**D**) Aggregates of zinc sulfide nanospheres, and archaea (a) with EPS structures (E) and bacteria (b), D1 (zoomed view) Nanospheres (N, see arrows) of micrometer-sized zinc sulfide particles/aggregates (sphalerite).

We observed precipitation of diverse nanometer- to micrometer-sized mineral particles, e.g. sphalerite (zinc sulfide), sulfur, iron colloids and iron sulfide, in microbiomes of sulfidic springs (Fig. 3). The precipitation of sphalerite in some microbiomes was also reported by Labrenz *et al*.^36^.

### Kinetics of metal removal by elicitation of EH8, EH10 and EH11

**Figure 4:**
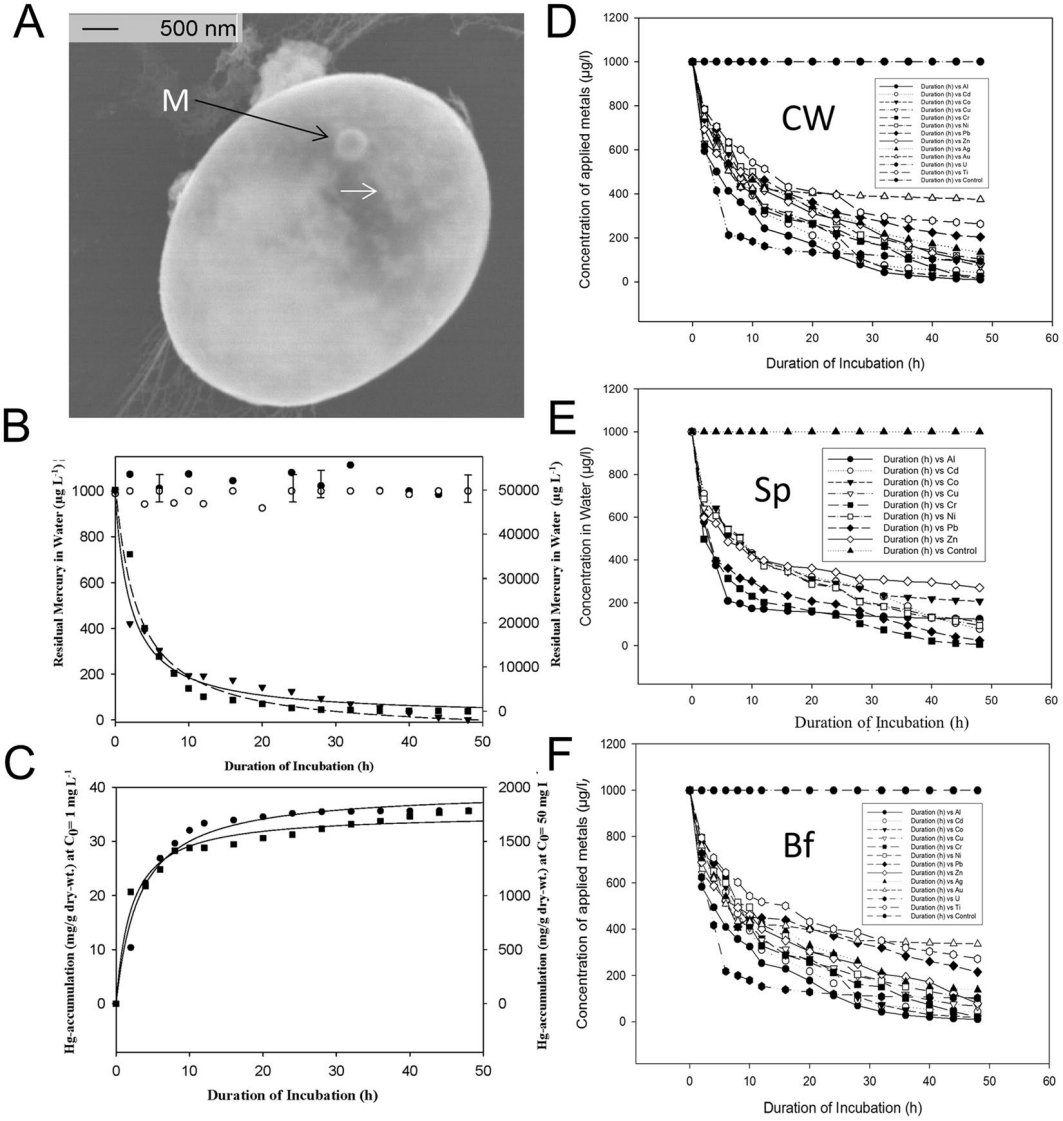
Kinetics of simultaneous metal ion removal. (**A**) Intracellular mercury accumulation (arrows) and deposition of metallic mercury nanospheres (M) by reduction of ionic mercury. (**B**) Similar kinetics of residual Hg after treatment of EH8’s activated sporangiospores with low (1 mg.L^−1^) and high (50 mg.L^−1^) concentrations^5^. (**C**) Similar kinetics of intracellular-fixed Hg at low (1 mg.L^−1^) and high (50 mg.L^−1^) concentrations. (**D-F**) Simultaneous removal of a metal mixture (>81–99% of Al, Cd, Co, Cr, Cu, Hg, Ni, Pb, U and Zn) by activated cell walls (CW, **D**), by activated spore mix (Sp, **E**) and by mixed microbiome (biofilm, Bf) grown on expanded clay spheres (**F**) of EH8, EH10 and EH11 (Fig. C.2) as well as enrichment of Au, Ag and Ti (Table 3). The fitted curve parameters with statistical significance are given in Table 2. Standard deviations of measurements (n = 3) at each data point were less than 5%.

The kinetics of metal removal depending on incubation time by fungal insoluble dead cell wall mix, spore mix and microbiome mix (EH8, EH10 and EH11) was compared with that of the control fungus (terrestrial *M. hiemalis* strain DSM2655, Fig. 4, Table 2). The hyper-accumulation of metals from contaminated water by fungal materials (see above) took place in two phases: an exponential phase (duration: ≤10 hours) and a slow phase (remaining duration: ≥38 hours) (Fig. 4B–F). In the exponential phase, more than 50% of metals applied were removed; the remaining metals were removed in the slow phase. It was demonstrated that the mixed dead cellwalls from EH8, EH10 and EH11 when compared to their live spore mix and live microbiome mix were similarly effective with respect to (1) a wide range of metals removed (Al, Cd. Co, Cu, Cr, Ni, Pb, Zn, Ag, Au, U, Ti) and (2) rate of metal accumulation from water. We demonstrated the high efficiency of metal removal and enrichment by mixed fungal dead cell walls by applying a metal mix of Al, Cd, Co, Cu, Cr, Ni, Pb, Zn, Ag, Au, and U, each at a concentration of 1,000 µg/l. A universal three-parameter rational function with y = (a + b*x)/(1 + c*x), whereby y = concentration of metal (µg/l) and x = duration of incubation (h) was shown to fit the metal remediation kinetics of all the metals applied optimally depending on incubation duration (h) (Table 2). Kinetics of residual mercury in water was inversely proportional to the amount of intracellular Hg fixed by EH8 independent of concentrations applied (1 mg.L^−1^, 50 mg.L^−1^), apparently due to high mercury tolerance and detoxification activity (Fig. 4B,C)^5^.

**Table 2:** Characteristic parameters of the generalized peak-fitting functions of insoluble dead cell walls (CW), spore mixtures (Sp) and cultivated fungal microbiomes (biofilms, Mb) describing highly efficient metal elimination by a combination of EH8, EH10 and EH11.

Metal	CW			Sp			Mb		
	Parameter	p≤	r^2^	Parameters	p≤	r^2^	Parameters	p≤	r^2^
Al	a = 1008.4159 b = −3.2817 c = 0.5618	0.0001 0.6744 0.0001	0.99	a = 1011.2979 b = −5.2787 c = 0.5561	0.0001 0.5797 0.0001	0.98*	a = 1011.5696 b = −5.5922 c = 0.5520	0.0001 0.5430 0.0001	0.98*
Cd	a = 991.8978 b = −15.4006 c = 0.2119	0.0001 0.0045 0.0001	0.98*	a = 994.2403 b = −7.9289 c = 0.2689	0.0001 0.0769 0.0001	0.99	a = 997.5382 b = 581.3458 c = 1.1636	0.0001 0.0004 0.0002	0.97
Co	a = 947.0998 b = 1.0934 c = 0.1012	0.0001 0.8530 0.0009	0.96*	a = 968.7509 b = 5.3617 c = 0.1183	0.0001 0.3410 0.0001	0.97	a = 944.6699 b = 0.1006 c = 0.0920	0.0001 0.9848 0.0007	0.96
Cu	a = 947.9398 b = -1.6486 c = 0.1453	0.0001 0.7235 0.0001	0.98	a = 938.9100 b = −0.0537 c = 0.1229	0.0001 0.9910 0.0001	0.99	a = 945.6613 b = −3.9292 c = 0.1349	0.0001 0.3609 0.0001	0.99*
Cr	a = 1007.0131 b = −12.5902 c = 0.4115	0.0001 0.0040 0.0001	0.99	a = 1006.7962 b = −15.4942 c = 0.3879	0.0001 0.0001 0.0001	1.00*	a = 1008.5005 b = −12.7635 c = 0.4033	0.0001 0.0093 0.0001	0.99*
Ni	a = 945.3009 b = 2.8120 c = 0.0958	0.0001 0.4924 0.0001	0.98	a = 947.2866 b = 0.7131 c = 0.1030	0.0001 0.8747 0.0001	0.98*	a = 951.5460 b = 2.0655 c = 0.1068	0.0001 0.6501 0.0001	0.98
Pb	a = 959.8348 b = 9.0128 c = 0.2611	0.0001 0.5030 0.0029	0.93*	a = 959.4407 b = 10.7195 c = 0.2548	0.0001 0.4267 0.0026	0.93	a = 959.8426 b = 10.8841 c = 0.2584	0.0001 0.4163 0.0023	0.93*
Zn	a = 1001.0351 b = 301.2079 c = 1.1359	0.0001 0.0001 0.0001	0.99	a = 1001.7014 b = 336.9611 c = 1.2772	0.0001 0.0026 0.0008	0.97	a = 1000.6658 b = 246.0606 c = 0.9843	0.0001 0.0001 0.0001	0.99*
Ag	a = 906.8907 b = −9.8880 c = 0.0487	0.0001 0.0053 0.0035	0.96	a = 913.6193 b = −5.8345 c = 0.0575	0.0001 0.1504 0.0029	0.96*	a = 907.2963 b = −7.9693 c = 0.0534	0.0001 0.0344 0.0033	0.96
Au	a = 906.4437 b = 9.8921 c = 0.0590	0.0001 0.2331 0.0104	0.94*	a = 924.8630 b = 23.8750 c = 0.0821	0.0001 0.0516 0.0052	0.94	a = 913.7804 b = 18.5878 c = 0.0756	0.0001 0.1083 0.0102	0.93
U	a = 1005.4774 b = 20.8824 c = 0.4439	0.0001 0.0033 0.0001	0.99*	a = 1006.6880 b = 34.1183 c = 0.4597	0.0001 0.0007 0.0001	0.99	a = 1004.7509 b = 19.2967 c = 0.3862	0.0001 0.1022 0.0001	0.97
Ti	a = 922.5146 b = 4.1861 c = 0.0788	0.0001 0.4496 0.0008	0.97*	a = 936.1490 b = 14.6439 c = 0.0994	0.0001 0.0531 0.0003	0.97	a = 942.3917 b = 15.7809 c = 0.1026	0.0001 0.0329 0.0002	0.97

Each row in the table represents an experimental subgroup, whereby a single ionic metal per subgroup was applied. The three parameters (a, b, c), the significance levels (p-values) by Student’s t-tests and the correlation coefficients (r^2^-values) are given for each ionic metal applied in a mixture (1,000 µg/l per metal) after fitting the residual concentrations (y) of each metal ion in water by the generalized function y = (a + b*x)/(1 + c*x) depending on incubation duration (x, 0–48 h). Standard deviations of measurements at each data point were less than 5%. The asterisk mark (*) indicates the highest elimination of the respective metal ion from the aqueous phase.

### Production of drinking water from metal-contaminated water by using successive fractionation and remediation biotechnology

**Figure 5:**
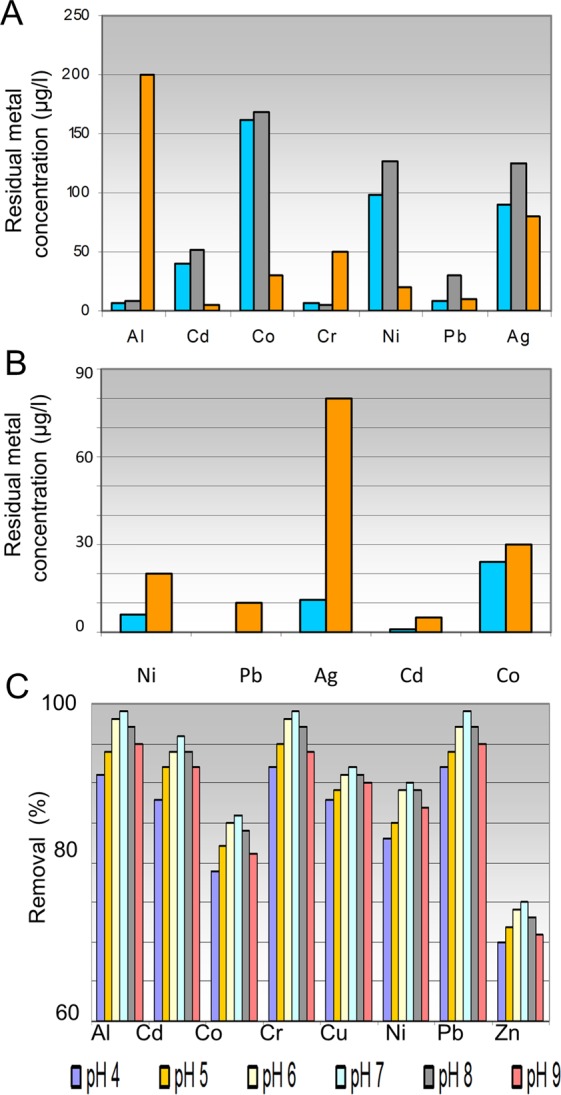
Demonstration of successive fractionation and remediation biotechnology for production of drinking water from multimetal contaminated water. Two-steps treatment of multimetal-contaminated water (1,000 µg/l per each metal in a metal mix) using dead insoluble cell walls (∼0.56 g fungal cell walls/L) is shown. (A) First step: Comparison of residual concentrations of ionic metals after 48 h treatment of contaminated water using insoluble cell walls from EH8, EH10 and EH11 (blue bar) or using fungal mixed grown microbiomes of EH8, EH10 and EH11 (grey bar) with the permitted concentrations (brown bar) for drinking water according to the German Drinking Water Ordinance. (B) Second step: Re-treatment of treated water from the first incubation using only dead insoluble cell wall mix led to reductions of concentrations of Ni, Ag, Cd and Co (blue bar) within 48 h even below the threshold values of the German Water Ordinance (brown bar) and (C) Removal (%) of metals (Al, Cd, Co, Cr, Cu, Ni, Pb, Zn) using mixed insoluble cell walls from EH8, EH10 and EH11 depending on pH 4–9.

The successive removal of various toxic metals by toxin-free insoluble cell walls of mixed grown EH8, EH10 and EH11 from water contaminated with 1,000 µg L^−1^ (Ag, Al, Cd, Co, Cr, Ni, Pb) is demonstrated for each metal in Fig. 5. Within 48 h the initial concentrations (each 1,000 µg/l) of aluminium and chromium decreased below the permitted threshold values for drinking water in the first step of fractionation e.g. following treatment of contaminated water with the insoluble mixed cell walls (Fig. 5A). After separation of cell wall materials in the first step, a re-treatment of water containing the residual metals Ni, Pb, Ag, Cd and Co with the same amount of mixed cell walls removed all of them, keeping the residual concentrations below the permitted levels of metals in drinking water according to the German Water Ordinance^37^ (Fig. 5B). The optimum metal removal by fungal cell walls occurred at neutral pH, although at pH below 7 or higher than 7 the percentage of metal removal decreased only slightly (Fig. 5C).

### Fractionation and biomining of metal ions by successive use of EH8, EH10, EH11 and mixed grown EH8-EH10-EH11

**Figure 6:**
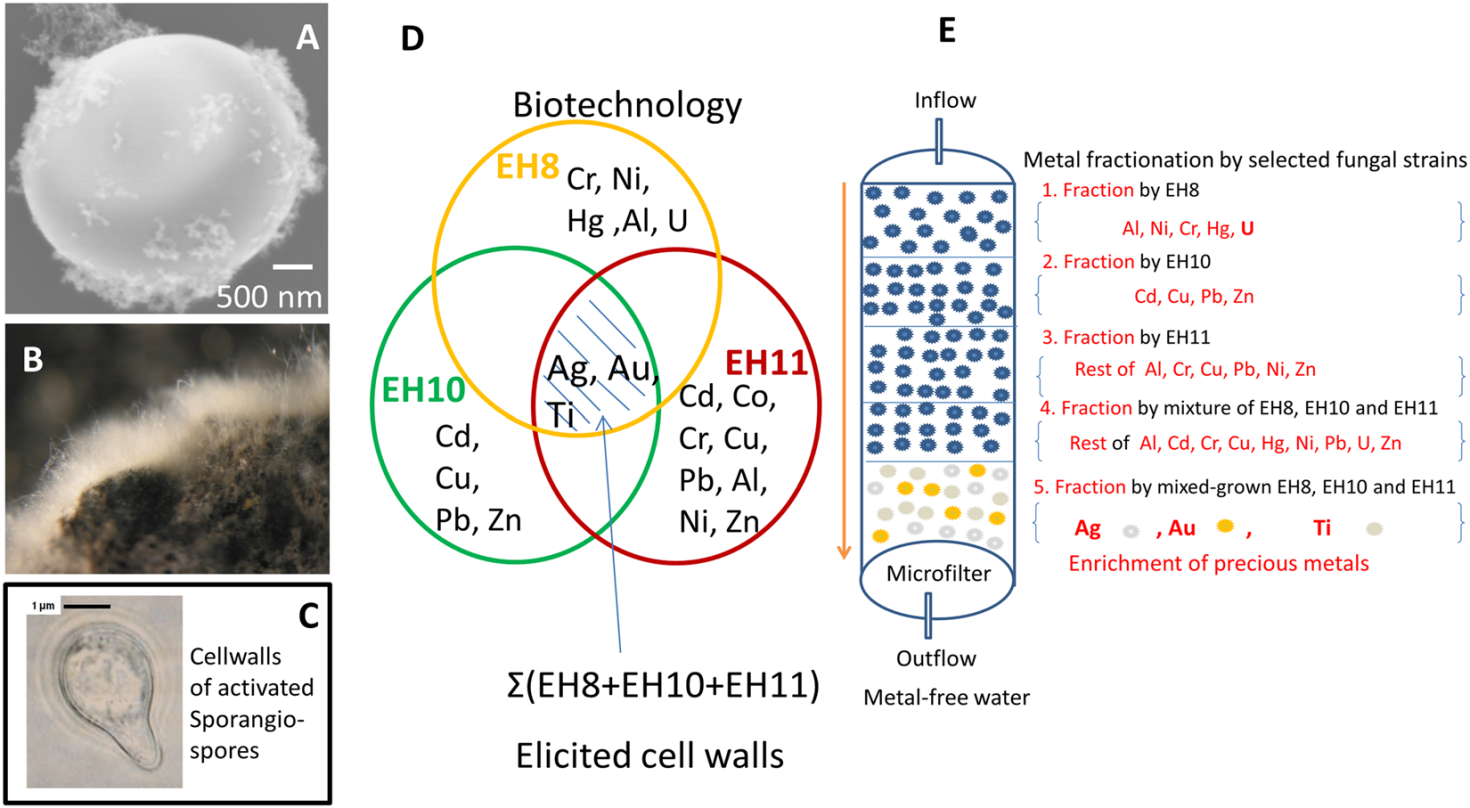
A new emerging biotechnology to enrich precious metals from diluted solutions. If grown together, the elicitation of EH8, EH10 and EH11 by interactions induces changes in chitinised cell walls (see supplement; **A**–**C**) capable of enriching precious metal ions like titanium, silver and gold (**D**), not following mathematical set theory for addition. (**A**) SEM of mixed grown activated germinating spores showing outer cell wall surface with bound nanometer-sized metal particles. (**B**) Stereo microscopic image showing elicited spores’ fungal microbiome grown on expanded clay spheres and (**C**) Solvent-killed and –purified insoluble dead cells/cell walls of mixed grown germinating activated sporangiospores, also to concentrate the precious metals as described. (**E**) Schematic set up for *ex situ* fractionation, bioremediation and biomining of ionic metals, especially precious metals, from multimetal-containing water phase.

Figure 6A–E show the concept of fractionation and biomining of metal ions based on experimental results. The first use of activated EH8 sporangiospores or microbiomes on expanded clay spheres will eliminate nearly all Hg(II) (99%), as well as some other metal ions as “fraction 1” from water by intracellular accumulation (Table S1)^5^. EH8 (Fig. 1A,B) of Marching spring (sporangiospores, microbiomes, cell walls) can retain Al (90%), Cr(III) (99%), Ni (86%), U (89%) as “fraction 2”^5^. EH10 (Fig. 1B) from Quarzitwerk spring biofilm will remove Cd (91%), Cu (85%), Pb (93%), and Zn (71%) as “fraction 3”. In contrast, aerobically cultivated anaerobic EH11 with its unusual spring-like hyphal adaptation (Fig. 1C.3–C.5) from the microbiome of methane-emitting salty sulfidic Künzing spring can adsorb Al, Cd, Co, Cr, Cu, Pb, Ni and Zn as “fraction 4”.

The characteristic ecological data and the glutathione S-transferase activities of the springs’ key organisms (EH8, EH10 and EH11), microbiomes and the terrestrial strain Mhh (DSM 2655) are shown for the comparison of the biosorption properties of single strain, mixed grown aquatic strains and microbiomes (Table 3). In contrast to the biomass-mixture of separately grown (non-elicited) spores, the biomass-mixtures of activated and mixed-grown (elicited) sporangiospores, cellwalls and microbiomes of EH8, EH10 and EH11 can fractionate and even enrich precious metal ions, e. g. U, Ag, Au, Ti (Tables 2, 3; Figs 2D, 4 and 6D).

**Table 3:** Ecological, biomarker (GST) and biosorption data of the strains EH8, EH10 and EH11.

A. Ecological Data						
**Aquatic Fungi** (Sulfidic-Sulfurous Springs)	**Mh EH10**		**Mh EH8**		**Mh EH 11**	
**Regional Occurrence/Spring**	Vicinity of Alps (Murnauer Moor) Quarzitwerk		West of Regensburg (Danube River Side) Marching		West of Passau (Danube River Side) Künzing	
**Water Age (years)**	114		140		>20,000	
**Start of microbiome formation** (2003)	May		March		Whole Year	
**Water temperature** (°C)	10.5		10.4		19.0	
**Hydrological Type**	Sulfide-Zinc-Hydrogen-carbonate (+Ca, +Mn)		Sulfide-Zinc-Hydrogen-Carbonate		Sulfide-Methane-Salt-Hydrogencarbonate	
**Sulfide content** (mg/L)	1.2		0.5		0.8	
**Mineral content** (mg/L)	728		537		1249	
**Redoxpotential** (E_h_, mV)	−106 to −97		−185 to −173		**−253 to −241**	
**Water quality** (Diatom-Saproby)	2.2		1.9		1.9	
**Trophy** (Diatom-index)	2.1		1.7		2.2	
**B. Biomarker (GST) Activity and Metal Biosorption Features**						
**GST-Activity** (nmol.min^-1^.mg^-1^)	Quarzitwerk		Marching		Künzing	
**Microbiomes:**	Cytosolic	Microsomal	Cytosolic	Microsomal	Cytosolic	Microsomal
IDNB	210.95	**9,109.62**	**15,486.36**	**5,161.41**	—	—
CDNB	1,347.78	**7,513.11**	**17,896.42**	4,328.00	—	—
DCNB	**24,256.05**	**14,789.03**	**13,803.01**	4,364.44	—	—
EPNP	**21,171.05**	**259,739.64**	**410,556.21**	**74,182.73**	—	—
Flourodifen	**10,229.79**	**54,066.00**	**21,178.58**	**55,418.01**	—	—
**Fungi:**						
IDNB (Mhh: 159.69/171.28)*	387.78	676.92	525.95	585.95	548.37	372.31
CDNB (Mhh: 153.03/188.77)*	174.20	399.20	553.21	655.23	358.31	412.54
DCNB (Mhh: 138.27/46.35)*	327.90	239.52	305.46	392.46	323.74	214.07
EPNP (Mhh: 4,701.20/6,303.42)*	**8,473.16**	**10,060.22**	**15,342.55**	**71,878.17**	**12,383.62**	**11,988.38**
Flourodifen (Mhh: 315.94/635.42)*	**1,294.71**	**1,313.52**	**680.00**	**2,856.84**	**1,146.61**	**1,519.26**
Association: Microbiome – Moos	No		Yes		No	
**Effective Metal Sorption (%)**	**EH10**	**Nm** ^**#**^	**EH8**	**Nm** ^**#**^	**EH11**	**Nm#**
Cd (Mhh**: 53.0%)	**92.0**	**93.8**	6.0	*95.5*	**81.0**	**96.6**
Co (Mhh**: 0.0%)	23.0	*93.6*	0.0	*99.3*	**81.0**	**99.5**
Cr (Mhh**: 90.5%)	38.0	***75.1***	**99.0**	**91.9**	**91.0**	**95.3**
Cu (Mhh**: 0.0%)	**89.0**	**90.8**	20.0	*93.6*	**83.0**	**94.8**
Pb (Mhh**: 96.0%)	**92.0**	**82.4**	18.0	*82.8*	**97.0**	**96.6**
Hg (Mhh**: 0.0%)	0.0	0.0	**99.0#**	**97.5**	0.0	0.0
Al (Mhh**: 0.0%)	18.0	*79.8*	**90.0**	**98.8**	**99.0**	**90.7**
Ni (Mhh**: 0.0%)	0.0	*91.8*	**86.0**	**95.1**	**82.0**	**99.7**
Zn (Mhh**: 24.8%)	**71.0**	**73.9**	46.0	48.1	**86.0**	**96.4**
U (Mhh**: 0.0%)	0.0	—	**89.0**	—	0.0	—
**Combination of elicited strains EH10, EH8 and EH11**						
New Sorptions (%)	**Ag 87**					
	**Au 61**					
	**Ti 75**					

The mean percentages of metal ion removal from water were calculated after measurements following applications of metal mixes with 1,000 µg/l per each metal to natural microbiomes (Nm, biofilms) and corresponding fungal cultures (F). The significant matched values of microbiomes and fungus of selected springs are shown in boldfont, whereas contrasting data are given in italics. Standard deviations of measurements (n = 3) at each data point were less than 5%. Abbreviations are marked: *Cytosolic/microsomal GST activity, **Mhh: *Mucor hiemalis f. hiemails* (DSM 2655, terrestrial strain) for comparison, # mostly intracellular accumulation, - not measured due to low amount of available materials, ^#^Matched values are in bold font; Sorptions by Nm (=natural microbiome with key fungus) > sorption by respective key fungus shown in italics are apparently due to other microbiome’s components, being not related to respective key fungus.

## Discussion

Harrison *et al*.^38^ suggested a multifactorial model by which biofilm populations can endure metal toxicity by a process of biodiversification. In contrast to the overall microbial- community role for the metal precipitation^38^, it was shown by our previous studies that if a key microorganism for metal removal exists in microbiomes, it can remove toxic metals simultaneously with high efficiency which is comparable to the microbiome itself (Table S1)^5,29^.

Adaptation and evolution of some key *M. hiemalis* microorganisms in hostile environments of cold sulfidic springs are sufficient to confer special metal resistance, hyper-metal accumulation and detoxification power that can give the whole microbiome selective advantages for survival and facilitate their microbial community members to select favourable successions in extreme microbial habitats (Tables S1, 2).

Hyper-metal accumulating *M. hiemalis* strains EH8, EH10 and EH11 from sulfidic spring water were tested for metal accumulation after inhibition/toxicity assays. Using this assay, they were found to be physiologically tolerant to each other without showing any antagonistic inhibitory effects, and hence used in combination for the development of new metal remediation biotechnology. For fungal metal binding and remediation, the cell wall components^11^ and their surfaces could play an important role. The cell wall of *M. hiemalis* consists of chitin (ca. 11%, Fig. S2), chitosan (32%), protein (5%) and phosphorus (1%)^11^, which are considered important for the determination of biotechnologically relevant features during growth and cell integrity^39^. In *M. hiemalis* MP/92/3/4 cell walls, the ratio of chitosan to chitin was ∼1: 0.84 (17.39% chitosan: 14.57% chitin, re-calculated), when the fungus was grown in adequate inorganic-N (sodium nitrate), whereas it decreased to ∼1:1.87 (8.7% chitosan: 16.3% chitin, re-calculated) when the fungus was grown in organic-N (peptone). Due to this ratio change with a concomitant decrease (about 21.8%, re-calculated) of Cr(III) biosorption, chitosan of *M. hiemalis* MP/92/3/4 cell walls is suggested to be involved in the biosorption of Cr(III)^11^. However, the increment of chitosan by ∼8.7% (re-calculated) is not in line with ~1.74% (re-calculated) decrease of chitin in the cell walls of inorganic-N grown *M. hiemalis* cells in comparison to the organic N-grown cells. Thus, chitosan cannot alone be responsible for the increment of biosorption by 27.91%, suggesting involvement of other functional groups, e.g. phosphate-group, in metal binding. In accordance with this interpretation, we found a strong P peak in aquatic *M. hiemalis* cell walls (Fig. 2B), where e. g. Al, Pb, Cd and Cr are adsorbed and precipitated as nanoparticles (Fig. 2B). The cell wall chemical composition of terrestrial *M. hiemalis* was variable, probably due to the influence of nutrition conditions, e.g. type of N-source^11^. Similarly, we also observed changes of cellular surface potential of aquatic *M. hiemalis* by varying nutritional conditions of growth medium (C-limitation; N-limitation; C-, and N-enriched medium; groundwater) (Fig. 2C). The zeta-potential of pure chitin showed enhancement of electro-negativity with increasing pH after pH ∼ 5.7 and reached the highest electronegativity ∼ −260 mV at pH ∼10.8 (data not shown). In contrast, the zeta potential of aquatic *M. hiemalis* spore surfaces at the lowest point is nearly double negative when compared to the zeta-potential of pure chitin, implying the involvement of other electronegative groups in the cell walls of aquatic *M. hiemalis* with twice the capacity for metal binding. The strong presence of a phosphorus peak at the location of precipitates of Al, Cd, Cr and Pb indicated high concentration of electronegative phosphate ligands for the complexation with positively charged metal cations at cell walls in aquatic *M. hiemalis* (Fig. 2B). Similarly, the deposition of Al and U cations into polyphosphate granules was also observed in the cell walls of *Anabaena cylindrica*^40^ and in *Citrobacter* sp^41^. The metal precipitation at the cell walls of germinated spores could be catalyzed by acid phosphatase activity by increasing release of phosphate anion ligand for the complexation with metal cation and its precipitation as inorganic insoluble metal phosphate^2^. The concentration of the phosphate ligand may also exceed the solubility product of the metal phosphate in proximity to nucleation sites on the cell surface^41^. Similarly, it is possible in *M. hiemalis* by sequential biosorption and precipitation of Cr(III)^11^. Additionally, the functional groups of chitin, chitosan and other cell wall components as well as changes of molecular configurations and associated high electro-negativity of aquatic *M. hiemalis* cell surface might be involved in the cellular metal precipitation process (Fig. 2B–D), apparently via regulation of cell wall integrity and signalling pathways^39^. The binding of positively charged heavy metal cations to the fungal cell walls was previously suggested to be due to the electrostatic attractions^42^. Electro-negativity of the cell surface apparently results from the occurrence of negatively charged (dissociated) functional groups like carboxylic-, amino-, hydroxyl- and phosphate groups^42,43^. Hydroxyl-, amino-, acetyl- and carbonyl groups can be also found on chitin and chitosan of fungal cell walls, but additionally the presence of carboxyl groups on the fungal cell surface was suggested to be important for the biosorption of Hg^2+^ (85.34%) and Co^2+^ (73.05%)^44^.

EDX microanalysis demonstrated that the removed metals were deposited as nanoparticles (approx. 50–100 nm) in clusters on the surface of the aquatic *M. hiemalis* cell walls (Fig. 2B). Nanometer- to micrometer-sized particles were deposited in the natural microbiomes of the sulfidic springs, probably due to aggregations induced by microbial interactions with metal cations^45^ (Fig. 3). Deposition of nanosized sphalerites (zinc sulfide) was shown to be mediated by the microbial community^36^. It was reported that microbes could change the orientation of metal ions in 3D space for the formation of nanospheres in the microbial community^45^. Here we demonstrate the use of a unique combination of EH8, EH10 and EH11 in producing diverse metal nanoparticles or nano-alloys, not only important for bioremediation, but perhaps also essential for e. g. biomedical (e.g. nano-contrasting substances in medical diagnosis), industrial (e.g. nano-catalysts) or biomining (e.g. selective enrichment) applications. Our discovery of this superior biotechnology for multiple nanometal particle and nano-alloy production is noteworthy as it supersedes biotechnologies focussed on single specialised fungi capable of producing only one particular metal nanoparticle^46^. For survival and additional resistance, the fungal microorganisms and microbiomes might have evolved the ability to interact their electronegative cell surfaces with toxic metal cations to produce non-toxic aggregates of nanospheres. SEM-EDX analysis showed intracellular accumulation of highly toxic ionic Hg and subsequent chemical reduction to metallic nanospheres by EH8’s live (germinating elongating) sporangiospores. Fixing of intracellular Hg was inversely proportional to the diminishing residual Hg in water phase with time, following a generalized kinetics function of intracellular Hg accumulation independent of applied concentrations (Fig. 4A–C)^5^.

All of the three fungal materials (mixed microbiomes, spore mix, dead mixed insoluble cells/cell walls) of combinations of EH8, EH10 and EH11 share a mutual mechanism of metal biosorption. They mainly use cell surfaces for simultaneous hyper-accumulation and precipitation of twelve tested metal ions (Fig. 5D), apparently due to appearance of extra special chemical and physical properties in their cell walls by elicitation that could not be revealed by the toxicity/inhibitory tests (Fig. 1). As the cell wall surfaces play a great role, the dead cells/cell wall skeletons irrespective of preparation method (autoclaving/sterilization, solvent killing) mainly contribute to the removal of most of the metals. However, calculations using curve-fitting point to preferential binding of Cd, Co, Pb, Au, U and Ti by solvent killed and solvent washed dead cells (cell wall skeletons) in juxtaposition to other metal ions (Table 2), supporting the view that killing of fungus increases biosorption of some metals^47^. Our calculations also showed preferential fixing of Al, Cr, Ni and Ag by the live germination-activated spores. Contrastingly, the fixing of Cu, Pb and Zn beside Al was rather preferred by the live fungal microbiomes (Table 2). However, high metal removal values of all of the tested materials showed maximum 31% variations among them depending on metal ion and material used. Noteworthy, the three metals Au, Ag and Ti were fractionated and enriched only by the above mentioned growth combination of EH8, EH10 and EH11 by elicitation, i.e. they were not hyper-accumulated by any individual EH8 or EH10 or EH11 or by any combination not grown together. The underlying mechanism of gaining additional metal removal/enrichment functions by their combination of EH8, EH10 and EH12 can be mainly explained on the basis of elicitation/adaptation of cell wall composition and functions, whereby increment of surface display of pertinent functional (electronegative) groups and orientation via molecular, biochemical and physiological interactions can be assumed to take place during cell wall biosynthesis of germinating/elongating EH8, EH10 and EH11 sporangiospores in a mix. Thus, such changes apparently result in changes of ionic interactions of electronegative functional groups of cell surface with metal cations, complexation reactions, chemical reduction and acceleration of metal precipitation processes. Analogously, Kotrba *et al*. showed four times more binding of Cd^2+^, but Cu^2+^ and Zn^2+^ in less amounts, to cell surface by genetically modifying the surface display of short metal binding peptides on *Escherichia coli*^18^. On the contrary, our fungal technology using EH8, EH10 and EH11 covers a wide range of metals for binding.

Inhibitory concentrations of copper or cobalt in the growth medium can also alter the cell wall’s metal binding properties of *Cunninghamella blakesleeana*^48^. The diverse metal removal efficiency of mixed cell walls (EH8, EH10 and EH11) in the range of 67% to >99% of initial concentrations (Fig. 2C) was similar to that of the corresponding spore mix and microbiome, but much higher than that of the terrestrial control fungus *M. hiemalis* strain DSM2655, which removed only Cd (53%), Cr (90.5%), Pb (96%) and Zn (24.8%). The maximum biosorption capacity of Cr(III) by the cell wall of terrestrial *M. hiemalis* MP/92/3/4 was given as 132 mg/g dry-weight, whereas the whole cells of terrestrial *M. hiemalis* MP/92/3/4 reached a maximum of 21 mg/g dry-weight^11^.

### The future

Naturally occurring EH8, EH10 and EH11 simultaneously removed nine toxic metals (Al, Cd, Co, Cu, Cr, Ni, Pb, Zn) and thereby also enriched four precious metals (U, Ti, Ag, Au) (Fig. 6A–E), a result that was elusive to date. For the first time it was demonstrated that if the physiologically tolerant (compatible) fungi were grown together, their respective functions became biologically elicited (enhanced) and acquired additional properties of Ti, Ag and Au enrichment, not following the mathematical union of the three sets of metal accumulations by EH8, EH10 and EH11 individually. This mixed fungal technology may help in promoting cheap biotechnology for bioremediation, biofractionation and biomining of metals. A new bioremediation technology with its wide coverage of toxic metals at high concentrations without any genetic modifications or pre-treatment may have high competitive advantages in securing a significant part of the multi-billion dollar remediation and biomining market^8^. Landfill leachate, mine drainage and waste water with toxic metal mixes can also be decontaminated by using column fillings of mixed grown EH8, EH10 and EH11. A step-wise treatment of multimetal-contaminated water using insoluble EH8, EH10 and EH11 cell wall mix can even deliver drinking water (Fig. 5A,B), which is just one of the application areas in which the biotechnological potential of aquatic *M. hiemalis* remains to be exploited. Even toxic Hg could be removed by live EH8 sporangiospores^5^. In contrast, most of the other microorganisms have so far failed to remediate a myriad of toxic metals simultaneously due to the extreme synergistic multimetal toxicity. As for example, simultaneous presence of Ni and Cd in solutions exerts toxicity to *Clostridium thermoaceticum* even at the low concentration of 1 mM^14^.

Some merits of this new biotechnology are worth mentioning:

1. Possibility of *ex-situ* and *in-situ* applications
2. Possible regeneration and recycling of biosorbents, e. g. by washing with 5 mM EDTA^48^,
3. Minimization of microbial waste by burning,
4. Possibility of multimetal nanospheres production for industrial and medical use, nearly independent of pH, e.g. only 10% efficiency loss was detected at pH 4 (Fig. 2B2, B2E),
5. Independence of growth from temperature variations in a wide range, enabling even an *in situ* treatment of a metal contaminated aquifer using fungal microbiomes on expanded clay down to near freezing temperature,
6. High safety assurance while using toxin-free fungal cell wall mixes for water purification,
7. Cheap biotechnology: even extracted fungal cell walls exhibited 2–3 times superior binding capacity for ionic metals compared to other materials used, thereby about 30% less cell wall biomass is required for the same efficiency of metal removal by using spore mixes or microbiomes. A calculation by Holan and Volesky^49^ showed at least double the metal removal capacity of insoluble fungal cell walls compared to marine algae on the basis of dry-weight.
8. Biological elicitation by the fungal strains’ mixed growth enhanced their ability to enrich precious metal ions more than the union of three sets of metal enrichments by the strain EH8, EH10 and EH11 individually, implying the biological set union has a system response greater than the corresponding mathematical set union.
9. In combination with *Phanerochaete chrysosporium*, aquatic *M. hiemalis* strains might also simultaneously remove many recalcitrant organic pollutants from their mixed contaminations with multiple metals in waste water^30–32^.

Last but not least, the presented results may inspire us to use these new strains EH8, EH10 and EH11 to concentrate and extract precious metals from oceans and seas at a low-cost, which was once tried in vain by the great German chemist F. Haber^50,51^. Later Necker also dreamt of extracting gold, silver and uranium from seas and oceans using emerging technology, but it was also found to be economically infeasible because of low concentrations (e.g. ~10 ng.L^−1^ gold and silver)^52^. A feasible low-cost method for biotechnological extraction of gold has awaited a break-through since the discovery that deposition of gold in ores was due to microbial activity^53^. Now we are on the verge of an economically feasible emerging biotechnology, simply showing nature’s power in special microorganisms and microbiomes of selected sulfidic springs to remediate multimetal-contaminated water and even to extract and concentrate some precious ionic metals from extremely diluted solutions present in lakes and oceans (Fig. 6). The use of insoluble fungal dead cell-wall fractions avoids the eco-toxicological risk and conflict arising from current legislation restricting the use of live fungi, microbiomes and/or genetically modified/engineered microorganisms^8^ for drinking water purification, bioremediation and/or biomining in the fields, as there is a risk of toxin release by live fungus. However, in presence of recalcitrant highly toxic organic substances mixed with toxic metals a combination of aquatic *M. hiemalis* with non-pathogenic *P. chyrsosporium* could be used^30^. The ability of mixed grown EH8, EH10 and EH11 to fractionate and enrich precious metal ions like U, Au, Ag and Ti from the aqueous phase opens a new possibility of simultaneous water purification, biofractionation and biomining of precious metals, even from highly diluted concentrations present in marine water. Thus, our results may be useful for the development of an efficient large-scale low-cost fungal biotechnology for the treatment of multimetal-contaminated industrial waste water, land-fill leachate and ground water^54,55^ and/or for biomining^8^ of valuable metals.

## Methods

### Heavy metal stock solutions

Heavy metal salts or solutions used were of analytical grade and obtained from commercial sources (Sigma-Aldrich-Fluka, Merck): Al(NO_3_)_3_.9H_2_O, AgNO_3_, AuCl_4_H aq., Cd(NO_3_)_2_.4H_2_O, Co(NO_3_)_2_.6H_2_O, Cr(NO_3_)_3_.9H_2_O, Cu(NO_3_)_2_.3H_2_O, HgCl_2_, Ni(NO_3_)_2_.6H_2_O, Pb(NO_3_)_2_, TiCl_3_, U(CH_3_COO)_2_.2H_2_O and Zi(NO_3_)_2_.6H_2_O. Stock solutions of required concentrations were prepared in ultra-pure de-ionized water below the solubility limit of the metal salts.

### Toxicity/inhibition assay

The tests of strain toxicity/inhibition i.e. strain compatibility based on strain morphology were carried out on malt-extract agar plates^5^ by inoculations of mycelium pieces^30,34^.

### Zeta-Potential of fungal spore surfaces

The surface charge (relative zeta-potential) of fungal spores at different pH was determined by measuring streaming current potential shift in the diffuse layer of fungal spore surfaces (electrostatic double layer model) after induction of a water stream by placing a current over the surface. For this purpose, the measuring electrode was fixed on a surface containing the fungal spores and the potential shift was detected using a particle charge detector Mütek PCD 03-pH (Mütek, Herrsching, Germany). Sporangiospores (2–5 ml spore suspensions) of aquatic *M. hiemalis* germinated in different nutrient liquid media (low-C; low-N; C-, and N-enriched media^35^; water control) were diluted to give final mean concentrations of about 4.4 × 10^7^ spores in 20 ml. The initial pH values of *M. hiemalis* spore dilutions were between 7.6 and 8.3. The titration of spore dilutions was started by using 0.1 M HClO_4_ in the acid phase and then by using 0.1 M NaOH in the alkaline phase. All the measurements were duplicated.

### Preparation of microbiomes, spore mix and dead insoluble mixed fungal cell walls

All the three strains, EH8, EH10 and EH11, were able to grow under aerobic conditions on malt extract-agar plates, although EH11 was also found to live under strictly anaerobic conditions in liquid cultures. The germinated spores of the strains were used as 1:1:1-mixture, as they were found to be physiologically compatible when challenged separately and against each other. Briefly, fungal sporangiospores (see results) from five malt extract-agar (2:1, wt/wt) plates of each strain were collected and enriched by filtration and centrifugation technique. The fungal spores were counted using a hemacytometer (Sigma) and image processing software (ProImage v.3.01, MicroMotion). Germination of sporangiospores (5×10^7^, EH8/EH10/EH11 = 1/1/1) was induced in 50 mL C-, N-enriched medium^35^ or on expanded clay spheres at 30 °C under shaking at 120 rpm. Active germinated spores (1–3 cell stages) were purified by repeated washing with PBS (pH 7.4) and centrifugation cycles, and then absorptions (A_270 nm_, A_650 nm_) of diluted suspensions were determined. Calibration curves for spore biomass (fresh- or dry-weight, mg) against absorption values (A_270 nm_, A_650 nm_) were calculated by regression analysis, and used to determine spore biomass, as for example, y (mg, spore biomass dry-wt.) = 12.26 + 8.096 * A_270 nm_, depending on absorbance. In contrast, microbiomes grown on expanded clay spheres in C-,N-enriched nutrient medium^35^ using the same mixed spore count of EH8, EH10 and EH11 were repeatedly purified by washing with PBS (7.4) without centrifugation prior to their use for our study.

Insoluble dead mixed fungal cells with cell wall skeletons (cell wall materials, see below) were prepared following a protocol for preparation of plant cell walls (Bligh and Dyer)^56^ with modifications. After aerobic germination of the three *Mucor hiemalis* strains, EH8, EH10 and EH11, (1: 1: 1, total 5×10^7^ sporangiospores per 50 ml water) in associations (tri-cultures), the fungal sporangiospores (1−3 cell stage) were pelleted by centrifugation. The live pellets were chemically killed by incubation in Bligh-Dyer solution^56^ for 4 h. After removal of supernatants, the insoluble dead cells with cell wall skeletons were successively washed with methanol-dichloromethane solution (3:1, v/v; 30 min), 1% SDS (12 h), 1 M NaCl (12 h), water (12 h) and acetone (30 min) to eliminate any remaining soluble cellular materials. The prepared dead insoluble fungal cell skeletons/cell walls were dried in a desiccator before use.

### Metal Removal Assay

Spore mix, mixed microbiomes and insoluble dead cell walls of EH8, EH10 and EH11 (see below) were used for the metal removal assays. Pre-germinated EH8, EH10 and EH11 sporangiospores (5×10^7^ spores, mixed in the 1:1:1 ratio) or mixed microbiomes or dead mixed cell walls (ca. 28 mg dry-weight) were suspended at least in triplicate in 50 ml ground water artificially contaminated either with a single heavy metal or with a heavy metal mix at a concentration of 1–50 mg/l, shaken continuously for 0–48 h at 120 rpm and 25 °C. After centrifugation at 4,000 × g (3 min) and after subsequent filtration (0.45 µm) and acidification (pH 1), with concentrated HCl or HNO_3_ and/or in aqueous solutions, and finally digestion of pellets (spores) in concentrated HNO_3_ and adjustment of pH to pH 1, the heavy metal contents in the supernatants were determined at element-specific wavelengths using an inductive coupled plasma detector (ICP, Model Liberty 200 A, Varian) as previously described^28^. The system was calibrated by using respectively 0, 1, 10, 100, 500 and 1000 µg/l heavy metal standards, whereby the calibration curves with 95% confidence intervals were calculated by linear regression analysis (r^2^ = 0.99). The standard deviations of measurements (n = 3) at each data point were less than 5%.

### Analysis by energy-dispersive X-ray (EDX)

After incubation with heavy metals, the fungal materials (spores) were separated by centrifugation, fixed with 1% glutaric aldehyde (15 min), then treated with 2% OsO_4_ in phosphate-bufferd saline (PBS, pH 7.4), washed and dehydrated in an increasing ethanol gradient (50%, 80%, 100%) in water. The uncut samples were sprayed with nanogold particles prior to observations with scanning electron microscopy (SEM, model JSM 630 F, Jeol) at 5–15 kV in the secondary electron mode. The sites of metal adsorption and precipitations were observed by using the SEM’s back scattering detector. A silicon peak detected is due to sample fixing on glass slides, traces of chloride and calcium peaks may also arise from the incubation medium. The elemental analysis of microbiomes, spores and cellwalls of EH8, EH10 and EH11 was carried out using eXL EDX-system (Oxford instruments). For this purpose, X-ray quanta were detected by a silicon detector, the data were analyzed using energy dispersive x-ray analysis software and the signals of element specific shells were resolved and annotated using Link-ISIS software^5^.

### Assay of pH-stability of metal sorptions by fungal materials

The capacity of heavy metal adsorption and accumulation by microbiomes depending on pH was studied in the pH range 3–7. As some metal salts might precipitate under alkaline conditions due to the formation of insoluble hydroxides, the data taken at pH higher than ∼7.5 are not taken into account. The fungal spores or microbiomes were incubated in water of selected pH containing heavy metals of various concentrations for 48 h. The pH of incubation water was monitored before and after experiment.

### Glutathione-S-transferase activity

The glutathione S-transferase activity of aquatic fungi was determined as described^28^.

### Statistical analysis

The statistical analysis of data was performed using the Student’s t-test and the Mann-Whitney U test^57^. The data for the curves were fitted by using the software Sigma Plot 8.0 for Windows (SPSS Inc., United States).

### Identification

All the strains were isolated and identified by comparison of morphology, mating behavior and/or molecular biological sequencing^5,28^.

### Strains

Strains EH8, EH10 and EH11 have been deposited in the German Collection of Microorganisms and Cell Cultures (DSMZ, Braunschweig, Germany) as strains DSM 16290, DSM 16291 and DSM 16292, respectively. The ITS1-5.8S – ITS2 sequences of plus- and minus-strands of aquatic *Mucor hiemalis* have been deposited at the Genbank under the accession numbers GU183689 and GU183690, respectively.

## Supplementary information

Dataset 1

## Data Availability

All data concerning the manuscript have been presented.
