# Supplementary material for: Multimetal bioremediation and biomining by a combination of new aquatic strains of Mucor hiemalis
Source: Sci Rep. 2019 Jul 16;9:10318. doi: 10.1038/s41598-019-46560-7 (PMC6635518; doi:10.1038/s41598-019-46560-7)
Supplement: Supplementary file 1 — Dataset 1 [file 41598_2019_46560_MOESM1_ESM.doc]

**Multimetal bioremediation and biomining by a combination of new aquatic strains of *Mucor hiemalis***

**Enamul Hoque* and Johannes Fritscher**

| Spring/Removal (%): Microbiome (NM), Fungus (F) | Hg  NM F | Cr  NM F | Al  NM F | Cd  NM F | Co  NM F | Cu  NM F | Ni  NM F | Pb  NM F | Zn  NM F |
| --- | --- | --- | --- | --- | --- | --- | --- | --- | --- |
| Teugen^a^ |  |  |  |  |  |  |  |  |  |
| Marching | **97.5 99.8** | **95.6 99.2** | **98.8 90.0** | 95.5 6.0 | 99.3 0 | 93.6 19.5 | **95.1 86.4** | 82.8 18.4 | 48.1 46.2 |
| Quarzitwerk | 0 0 | 56.7 38.3 | 79.8 18.0 | **93.8 91.3** | 93.6 23.6 | **90.8 88.5** | 91.8 0.0 | **82.4 91.7** | **73.9 71.3** |
| Künzing | 0 0 | **92.4 89.5** | **90.7 98.9** | **96.6 80.3** | **99.5 80.6** | **94.8 87.2** | **99.7 82.1** | **96.6 97.1** | **86.4 83.6** |

**Table S1. Biosorption of ionic metals and metalloids by Marching´s, Quarzitwerk´s and Künzing´s microbiomes (biofilms) and their *Mucor hiemalis* strains.** The matched values of metal removal by natural microbiomes (NM) and corresponding *Mucor hiemalis* strain (F) are shown in bold font. Mean percentages of ionic metal removal from water are separately given for each metal cation (1,000 µg/L) applied in a mixture to natural microbiomes and corresponding fungal cultures of Marching (F: EH8), Quarzitwerk (F: EH10) and Künzing (F: EH11) springs in comparison to control spring Teugn (F: EH7).* Standard deviations of measurements (n=3) at each data point were less than 5%.


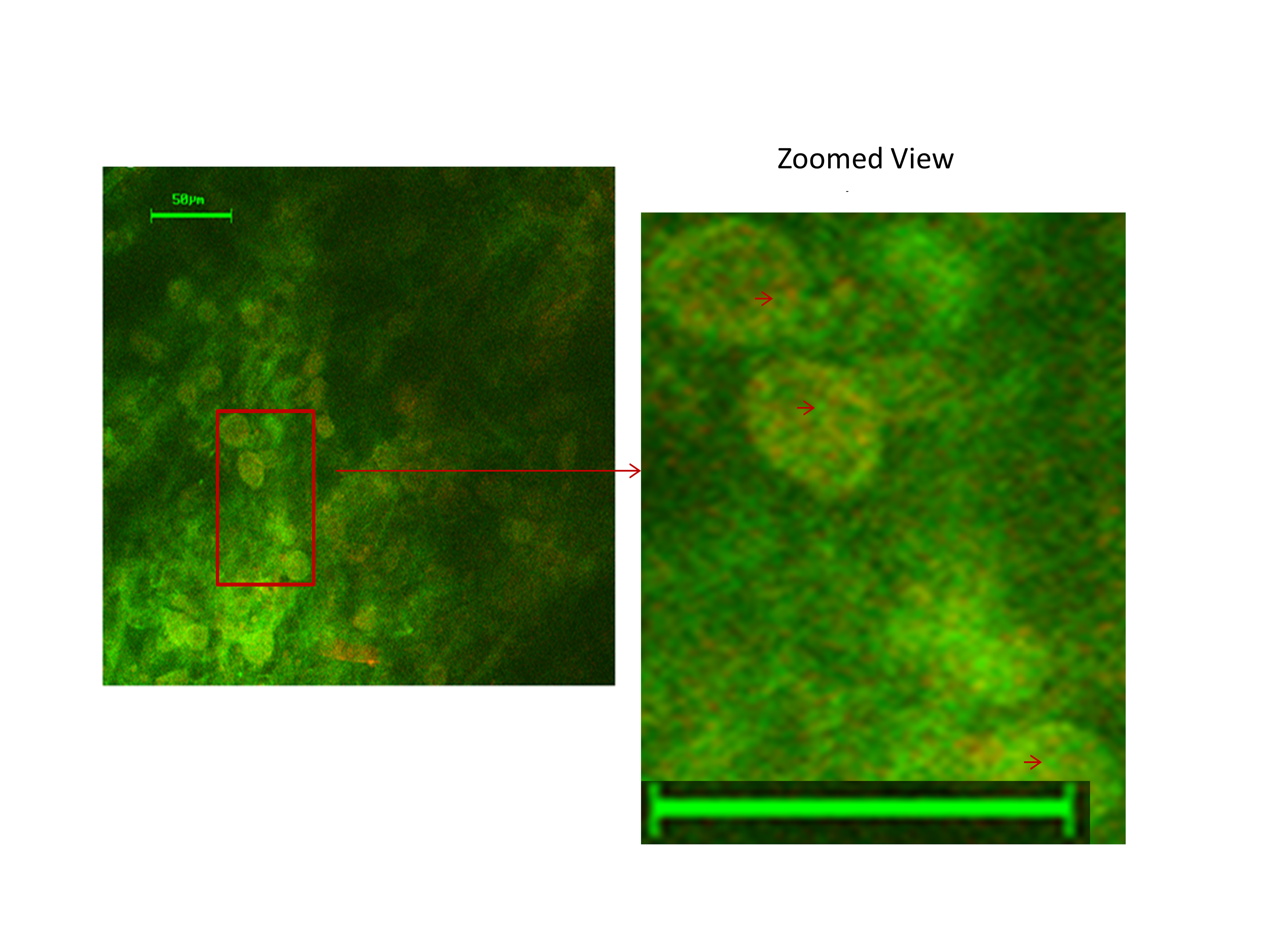


**Figure S2.** **Visualization of chitin on viable germinating sporangiospores of aquatic *Mucor hiemalis* from cold sulfidic springs by anti-chitin fluorescein antibody staining.** Note formation and uniform distribution of spherical fluorescein-labelled chitin binding proteins (see red arrows in zoomed view), apparently by reduction, on electronegative reducing chitinised centers of cell walls and in the vicinity, analogous to the formation of metallic nanospheres of bound metal cations by reduction^5^ on the cell wall surface (Fig. 2B2,2B2E) and in its vicinity as well as intracellularly (Fig. 4A;see S3 Methods below).

**Methods S3.**

**Chitin staining by specific recombinant chitin-binding protein.** Two drops of periodic acid (5%) were added to cover the viable germinating fungal specimen „sporangiospores“ in a hybridization-well plastic chamber fixed on a slide (Sigma, Germany) and incubated for 10 minutes at room temperature. Following periodic acid treatment the fungal specimen was rinsed three times with de-mineralized water and excess water was removed. Two drops of bovine serum albumin (5%) were added to cover the entire specimen and incubated for 10 minutes. Following this incubation, two drops of recombinant chitin-binding proteins conjugated to fluorescein N-hydroxylsuccinimide (Fungalase^TM^ conjugate, Anomeric^TM^ Inc., USA) were used to cover the entire specimen and incubated for 30 minutes at room temperature in a dark humidified chamber. After reaction the specimen was rinsed three times with several drops of phosphate-buffered saline (PBS) (20 mM phosphate, 150 mM NaCl) (pH 7.4) and the excess fluid around the specimen was removed. A laser-scanning fluorescence microscopy system (Zeiss, Model 11, Germany) was set at λ_excitation_ = 488 nm and 543 nm, and λ_emission_ = 510−565 nm and > 590 nm in order to visualize fluorescein-labelled chitin on the fungal cell wall surface.
